# Physiological and Pathological Role of mTOR Signaling in Astrocytes

**DOI:** 10.1007/s11064-024-04306-6

**Published:** 2024-12-09

**Authors:** Luise Hochmuth, Johannes Hirrlinger

**Affiliations:** 1 Carl-Ludwig-Institute for Physiology, Faculty of Medicine, University of Leipzig, D- 04103 Leipzig, Germany; 2 Department of Neurogenetics, Max-Planck-Institute for Multidisciplinary Sciences, D- 37075 Göttingen, Germany

**Keywords:** mTOR, Astrocyte, Glutamate, Mitochondria

## Abstract

The mammalian target of rapamycin (mTOR) signaling pathway is one of the key regulators of cellular energy metabolism. It senses diverse alterations in the extracellular environment such as availability of nutrients and growth factors, and mediates the corresponding intracellular response. In the brain, astrocytes crucially contribute to energy and neurotransmitter metabolism, and numerous other functions. However, the relevance of physiological, astrocytic mTOR signaling in maintaining brain homeostasis and function is not well understood. Pathophysiological mTOR signaling is involved in manifold diseases in the central nervous system and most of the knowledge about astrocytic mTOR signaling has been derived from observations on these disorders. Dysregulation of the mTOR signaling pathway impairs important functions of astrocytes including neurotransmitter uptake and -signaling as well as energy metabolism. Some of these alterations could trigger neuropathological conditions such as epilepsy. This review focuses on how mTOR signaling regulates properties of astrocytes, and how these signaling events might contribute to the physiological function of the brain.

## Introduction

Astrocytes are a type of glial cells in the central nervous system (CNS), which contribute to many functions of the brain including the maintenance of energy homeostasis, synaptogenesis, the regulation of the blood-brain-barrier, learning processes, control of behavior, and others [1–5]. Astrocytes form neuron-glia assemblies with their interaction partners in the local circuitry. They are recognized as a highly heterogeneous cell population, differing in morphology, metabolism, and function depending on their location in the brain, but also during different disease states [6–12]. Therefore, it is hardly surprising that disturbances in astrocytes’ function, and specifically astrocyte metabolism, are associated with a variety of brain pathologies.

The metabolism of astrocytes and neurons is interconnected with both types of cells expressing specific metabolic properties [13]. Glucose, provided by the blood, is the main energy substrate for the brain. Astrocytes and neurons can take up glucose from the extracellular space via glucose transporter 1 (GLUT1) and GLUT3, respectively [13–15]. In astrocytes, glucose can be metabolized via glycolysis, the pentose phosphate pathway (PPP) or stored as glycogen [13, 16, 17]. While there is an ongoing debate, whether lactate produced by astrocytes is essential for covering neuronal energy requirements [13, 18–20], there is evidence that astrocytic lactate production is modulated by adrenergic, purinergic and endocannabinoid signaling and contributes to memory formation [4, 21–24]. In astrocytes, pyruvate produced during glycolysis is transported into mitochondria and undergoes oxidative decarboxylation thereby fueling mitochondrial ATP production [13]. In addition, astrocytes express pyruvate carboxylase, a mitochondrial enzyme catalyzing the anaplerotic reaction from pyruvate to oxaloacetate [25–28]. Furthermore, astrocytes release glutathione to support neuronal antioxidative defense [29, 30]. Another important aspect of astrocytic metabolism is the glutamate/γ-aminobutyric acid (GABA)-glutamine cycle [31]. Astrocytes take up glutamate via excitatory amino acid transporters (EAAT1, also called GLAST) and EAAT2 (also called Glt-1) and recycle it back to neurons as glutamine. Glutamine is also used by GABAergic neurons as precursor for GABA, while GABA released from these neurons can be taken up and metabolized by astrocytes [32]. Furthermore, glutamate is an important gliotransmitter released by astrocytes to signal to neurons, thereby, for instance, influencing synaptic efficiency, plasticity, the synchronization of neurons, neural circuit activity and network states [33–36]. Therefore, as disturbances in astrocytic metabolism can have far-reaching consequences for the CNS, there needs to be a tight control and regulation of astrocytic metabolism in the context of the local neuron-glia assemblies.

**Fig. 1:**
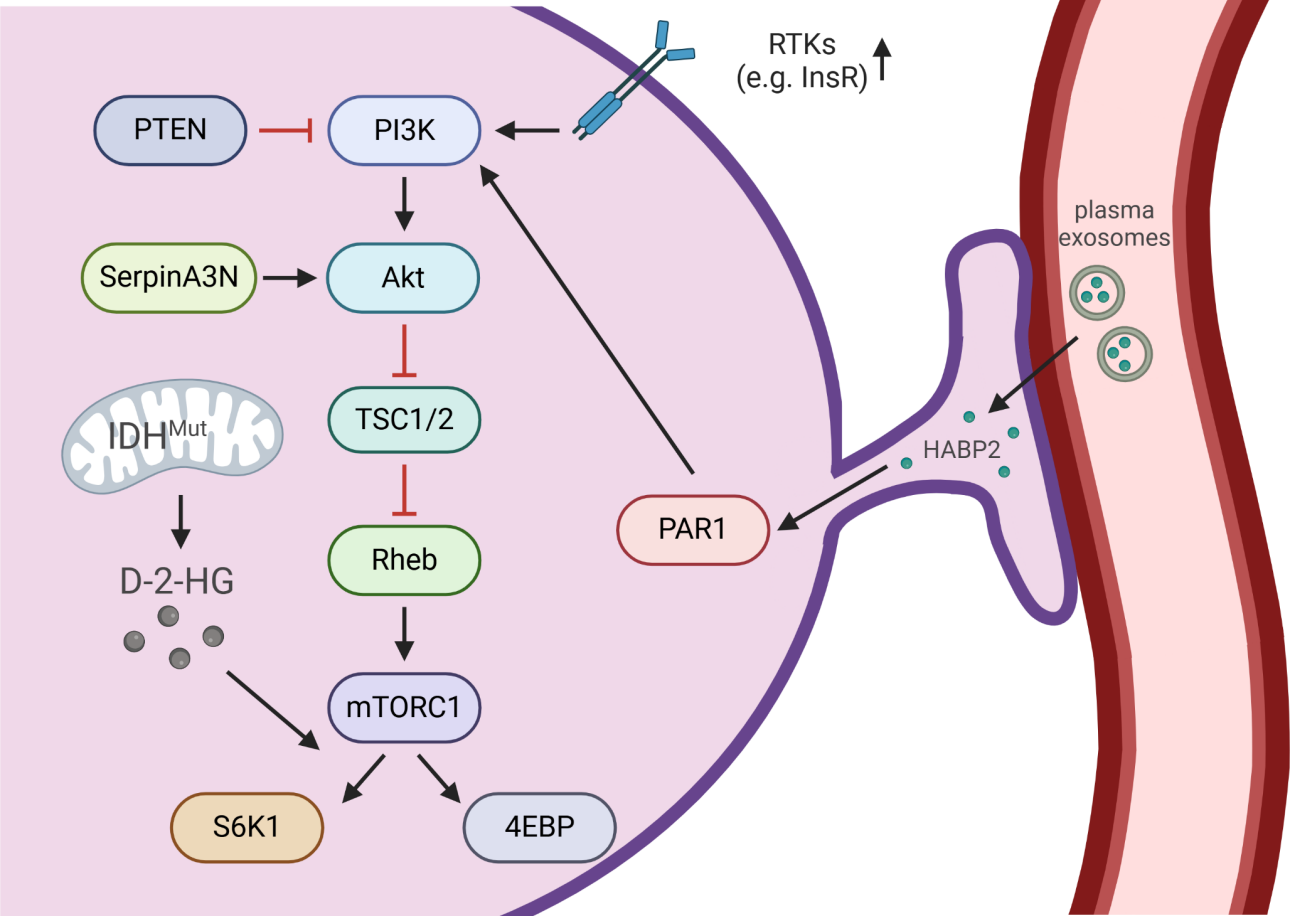
**The mTOR signaling pathway in astrocytes (simplified scheme).** mTOR is activated by a signaling cascade which includes positive and negative regulators. Downstream of mTOR, important targets like 4EBP and the kinase S6K1 are activated. Black arrows indicate activation, while red T-shaped lines indicate inhibitory interactions. 4EBP - eIF4E Binding Protein, Akt - Protein kinase B, D-2-HG - D-2-Hydroxyglutarate, HABP2 - Hyaluronan-binding protein 2, IDH– Isocitrate dehydrogenase, InsR– Insulin receptor, mTORC1– mammalian target of rapamycin complex 1, Mut - mutation, PAR1 - Protease-activated receptor 1, PI3K - Phosphoinositide 3-kinase, PTEN - Phosphatase and tensin homolog, Rheb - Ras homolog enriched in brain, RTKs - Receptor tyrosine kinases, S6K1 - Ribosomal protein S6 kinase beta-1, TSC1/2 - Tuberous sclerosis complex 1 / 2. Created in BioRender. Hirrlinger, J. (2024) https://BioRender.com/a64w464

The mammalian target of rapamycin (mTOR) signaling pathway is a crucial mediator between nutritional signals and an appropriate cellular response, which regulates cell survival, autophagy and metabolism. mTOR is a serine/threonine protein kinase that constitutes the catalytic subunit of two distinct protein complexes. mTOR complex 1 (mTORC1) consists of the proteins mTOR, regulatory protein associated with mTOR (Raptor), and mammalian lethal with Sec13 protein 8 (mLST8), among others, and is responsible for regulating cell growth according to nutritional signals such as oxygen, energy, amino acids and stress [37]. mTORC1 regulates protein synthesis, protein degradation, autophagy, as well as glucose, lipid, amino acid and nucleotide metabolism [38–40]. Important downstream targets of mTORC1 are p70S6 Kinase 1 (S6K1) and eIF4E Binding Protein (4EBP) (Fig. 1). Furthermore, transcription factors including activating transcription factor 4 (ATF4), sterol regulatory element binding protein (SREBP), and hypoxia inducible factor 1, α-subunit (HIF1α), are targets of regulation by mTORC1 [38–40]. In contrast to mTORC1, mTOR complex 2 (mTORC2) consists of mTOR, mLST8, and rapamycin insensitive companion of mTOR (Rictor), among others [41]. Therefore, it is hardly affected by the common mTORC1 inhibitor rapamycin. mTORC2 is mainly activated by growth factors and regulates cell proliferation and survival through phosphorylation of protein kinase B (Akt) and several members of the AGC family of protein kinases (protein kinase A, G, and C; PKA/PKG/PKC) [38]. An important upstream regulator of mTORC1 signaling are the tuberous sclerosis complexes 1 (TSC1) and 2, which inhibit the activity of mTORC1 (Fig. 1). A detailed description of the highly complex signaling network involving mTORC1 and mTORC2 is beyond the scope of this review; but can be found in recent reviews [38–40].

Although mTOR is well known as key regulator of energy metabolism, its contribution to the control of carbohydrate metabolism is not completely understood [39]. It is assumed that both mTOR complexes can detect glucose levels [39, 42, 43]. Chronic mTORC1 inhibition by rapamycin, as well as the disruption of mTORC2 in various tissues, leads to insulin resistance [3, 44–46]. Hyperactivation of the mTOR signaling pathway via inhibition of the TSC1/2 complexes alters the expression of glucose-6-phosphate dehydrogenase (G6PD) and GLUT1 [47]. Furthermore, both mTOR complexes control the carbon flux into the PPP mainly by increased expression of G6PD and ribose-5-phosphate isomerase A (regulated by mTORC1) as well as by phosphorylation of transketolase via protein kinase B (AKT; regulated by mTORC2) [47–49]. The mTOR signaling pathway thereby controls the production of NADPH, purines, and pyrimidines, which are important for lipid and nucleic acid synthesis and thus also influences cell proliferation [39]. Another important aspect of mTOR signaling is the regulation of protein synthesis and amino acid metabolism. Activation of the mTOR downstream targets 4EBP and the kinase S6K1 cause the phosphorylation of various eukaryotic initiation factors, which play a key role during translation initiation [50–52]. Furthermore, inhibition of mTORC1 downregulates the expression of asparagine synthetase and neutral amino acid transporters, while inhibition of mTORC2 enhances the release of glutamate and cysteine uptake [53, 54].

Dysregulation of the mTOR signaling pathway has serious consequences for cell and tissue homeostasis and contributes decisively to a variety of diseases in the CNS. Neurological disorders caused by genetic defects within the mTOR pathway are called mTORopathies and are characterized by malformations in the cerebral cortex, epileptogenesis, cognitive impairment and autism [55]. mTOR hyperactivation is a common feature of several brain diseases and correlates with the severity of the diseases [56]. For instance, tuberous sclerosis complex (TSC) is a typical mTORopathy, which is caused by loss of function mutations of TSC1 or TSC2 resulting in hyperactivation of the mTOR signaling cascade (Fig. 1). Furthermore, dysregulated mTOR activity promotes tumor growth of subependymal giant cell astrocytomas (SEGAs) in TSC as well as in cells from glioblastoma multiforme (GBM) [57–59].

## Mechanisms and Effects of mTOR (over-) Activation in Astrocytes

Important insights in the function and relevance of mTOR signaling has been obtained in conditions with dysregulation of this signaling pathway in astrocytes, which can result from a variety of changes within the regulatory cascade and from peripheral influences. In the context of ischemic stroke, peripheral blood-derived exosomes containing hyaluronan-binding protein 2 (HABP2) promote the expression of protease-activated receptors 1 (PAR1) in astrocytes causing activation of the phosphoinositide 3-kinase (PI3K) / AKT / mTOR pathway (Fig. 1) [60]. Another molecule that is upregulated in astrocytes and neurons during ischemic strokes is SerpinA3N, which induces increased phosphorylation of AKT and mTOR in vitro and in vivo [61].

Furthermore, the mTOR signaling pathway plays an important role during embryonic development [62, 63]. It is therefore not surprising that disturbances in this signaling pathway affect brain development and cell maturation. In the disease TSC, mutations of TSC1 or TSC2 result in loss of TSC1/2 function and, thereby, hyperactivation of mTOR signaling (Fig. 1). This genetic dysregulation of mTOR signaling causes changes of the structure of the whole brain, as well as of the cellular morphology of both neurons and astrocytes. During cortical development, focal lesions, called “tubers”, are formed in TSC brains, which are characterized by cortical dyslamination and the presence of different types of (diseased) cells, like dysmorphic neurons, giant cells as well as reactive astrocytes. Furthermore, patients develop SEGAs and epilepsy [59, 64–66]. The activation of mTOR also alters the morphology and function of astrocytes so that they phenotypically resemble an immature cell type [67], characterized as large and vimentin positive cells, which express high GluR4/GluR2 and high NR2A/NR2C ratios in their α-amino-3-hydroxy-5-methyl-4-isoxazolepropionic acid type glutamate receptors (AMPAR), and N-methyl-D-aspartate type glutamate receptors (NMDAR), respectively [68–70].

Additional insight on potential regulatory mechanisms affected by mTOR signaling in the brain is provided by observations on brain tumors, like GBM, which is one of the most common and most severe brain cancer types [71]. It has to be noted that GBMs are highly heterogenous regarding many aspects, including genetic mutations, expression levels of tumor associated genes, as well as cell composition, containing cells with astrocytic, oligodendrocytic, neural progenitor, and mesenchymal features [72, 73]. Therefore, observations on GBM cannot be directly extrapolated on physiological functions of mTOR signaling in astrocytes, but may only hint to potential functions. In many cases of GBM, an increased activity of mTOR signaling results in elevated cell proliferation and cell survival, with a simultaneous reduction in apoptosis, thereby promoting tumorigenesis and treatment resistance [57, 58]. However, the causes of PI3K / AKT / mTOR pathway hyperactivation in the context of GBM are manifold. Various mutations such as loss of function mutations of the tumor suppressor “phosphatase and tensin homolog” (PTEN), which acts as a negative regulator of PI3K, or mutations of receptor tyrosine kinases such as platelet-derived growth factor receptor (PDGFR) and epidermal growth factor receptor (EGFR), can result in permanent activation of PI3K, an upstream positive regulator of mTOR (Fig. 1) [57, 74]. In addition, in some gliomas an activating mutation of isocitrate dehydrogenase (IDH) results in overproduction of (D)-2-hydroxyglutarate (D-2-HG), which activates mTOR signaling [75, 76].

In summary, various factors are known to induce activation of the mTOR signaling pathway in astrocytes. However, also other, so far unknown, mechanisms most likely result in dysregulated mTOR signaling in the context of neurological disorders. Furthermore, much less is known about the physiological regulation, activation, and relevance of this crucial signaling pathway in the healthy brain.

## mTOR Signaling Regulates Astrocytic Neurotransmitter Signaling and Metabolism

**Fig. 2:**
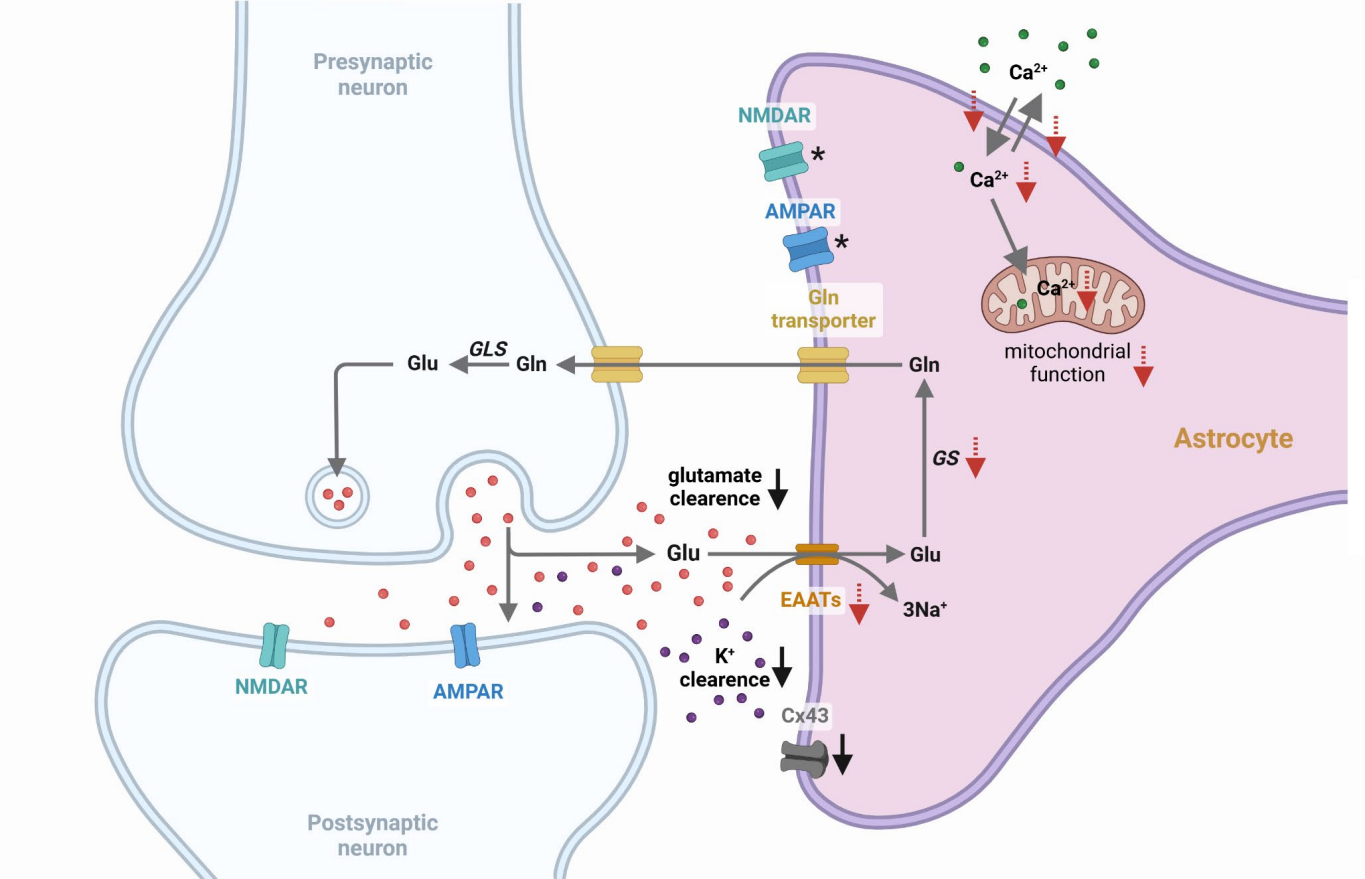
**Effects of activated mTOR signaling in astrocytes.** Black arrows show alterations due to increased phosphorylation of S6K1 or which are reversible using rapamycin. Red dashed arrows show alterations in astrocytes derived from TSC tissue. Asterisks show changes in receptor composition due to activated mTOR signaling. AMPAR - α-amino-3-hydroxy-5-methyl-4-isoxazolepropionic acid receptor, Cx43 - connexin 43, EAAT - excitatory amino acid transporter, GLS– Glutaminase, GS - Glutamine synthetase, NMDAR - N-methyl-D-aspartate receptor. Created in BioRender. Hirrlinger, J. (2024) https://BioRender.com/z22t421

Activation of mTOR signaling, in particular increased phosphorylation of S6K1, alters the expression and composition of various receptors and transporters involved in glutamate signaling. It reduces the expression of the glutamate transporters EAAT1 and EAAT2 in astrocytes (Fig. 2), restraining glutamate clearance from the synaptic cleft [69, 77–79]. This downregulation can be reversed by the mTOR inhibitor rapamycin, which blocks the phosphorylation and activation of S6K1. Consistently, in human TSC derived astrocytes a decreased expression of EAAT1, EAAT2, glutamine synthetase (GS) and the GluR1 subunit of AMPARs was described; however, activation of S6K1 was not directly confirmed [67]. Vice versa, rapamycin increases the expression of EAAT2 in TSC1 knockout and control mice, thereby reducing extracellular glutamate levels [67, 80]. Astrocytes from human cortical TSC tissue express AMPA- and NMDA-glutamate receptors with subunit compositions typical for immature astrocytes from midgestational brains (Fig. 2) [69]. Furthermore, different subtypes of astrocytes were described in TSC patients, which differ greatly in terms of mTOR activation, morphology and expression patterns [69]. However, whether these effects are a direct consequence of mTOR dysregulation in the astrocyte itself, or whether mTOR activation in other types of cells, e.g. neurons, induce signals which then affect astrocytes has not been unequivocally resolved yet and remains to be determined for many cases.

In addition to glutamate uptake and glutamate receptor signaling, mTOR also influences potassium clearance by downregulating the expression of the astrocytic gap junction protein connexin 43 (Cx43), thereby affecting astrocytic gap junction coupling (Fig. 2) [81]. In mice with genetic deletion of Tsc1 in astrocytes, Cx43 expression is reduced, resulting in increased extracellular concentration of potassium. This phenotype is reversed by treatment with rapamycin, suggesting that it is caused by activation of mTOR signaling [81].

Using astrocytes derived from human TSC patients, an impact of the mTOR pathway on astrocytic calcium signaling and mitochondrial metabolism was suggested (Fig. 2); however, mTOR activation or S6K1 phosphorylation was not directly confirmed in these astrocytes, but assumed from the TSC mutations [82]. This study showed transcriptomic and proteomic alterations in signaling pathways involved in calcium regulation, calcium-dependent neurotransmitter signaling, cellular respiration and mitochondrial metabolism [82]. Furthermore, cultured primary astrocytes from TSC patients exhibit lower calcium uptake and release as well as decreased basal cytosolic and mitochondrial calcium levels [82]. Mitochondrial dysfunction in TSC astrocytes was characterized by depolarization of mitochondrial membrane potential, decreased calcium exchange, reduced respiratory capacity and oxygen consumption rate as well as altered mitochondrial morphology, including mitochondrial fragmentation and structural remodeling of mitochondria [82]. Whether these observations reflect physiological regulations of astrocytic metabolism by mTOR or are caused by long-term adaptations to the genetic defect in TSC astrocytes remains to be established.

In summary, in astrocytes, mTOR signaling affects several aspects of glutamate signaling, synaptic glutamate clearance, glutamate metabolism, potassium buffering, calcium signaling as well as mitochondrial metabolism (Fig. 2). Dysregulation of these functions of astrocytes by pathological activation of mTOR could explain the epileptogenesis in several diseases associated with impaired mTOR signaling. However, it has been discussed whether seizures are a result of mTOR activation or if they induce mTOR activity [70]. While in a kainic acid seizure mouse model evidence has been obtained indicating seizures promote mTOR activity [83], induction of seizures by mTOR dysregulation is also a likely scenario. Therefore, further studies on the impact of astrocytic mTOR signaling in synaptic function are needed and could pave the way for identifying new targets for therapies against epilepsy.

## Mutual Influence of mTOR Activity between Astrocytes and Neurons

As described above, numerous studies provide evidence for the function and relevance of mTOR signaling in astrocytes. However, the brain is a complex organ with a complex interplay of various cell types including astrocytes, neurons, oligodendrocytes, microglial cells, pericytes, endothelial cells and others. Most likely, all of these cell types use the mTOR signaling pathway for their own needs with cell type-specific properties. Therefore, in some scenarios, for instance in models with general mTOR activation due to TSC mutations, it is not obvious whether alterations in morphology and functions of astrocytes are a result of mTOR dysregulation within astrocytes themselves or if mTOR dysregulation in the surrounding cells and tissues affect astrocytes. Furthermore, astrocytic homeostasis depends on neuronal mTOR activity. Deletion of Raptor in murine neurons inhibits mTORC1 signaling and reduces neuronal fibroblast growth factor 2 release, which causes enhanced astrogliosis [84]. This underlines the influence of the signaling status of the surrounding tissue on the properties of astrocytes and complicates the assessment of direct and indirect metabolic effects in diseases such as TSC. On the other hand, astrocytes activated by oxygen-glucose-deprivation / reoxygenation directly influence mTOR signaling in neurons via the release of exosomes containing nicotinamide phosphoribosyltransferase [85]. The resulting increase in neuronal autophagy improves the outcome of neuronal injury during acute ischemic stroke [85]. Taken together, mTOR signaling in astrocytes is part of a complex signaling network within these cells, but also closely interconnected with signaling and functions of the other cell types of the brain. However, these interdependencies and their functional relevance are currently not well understood and future studies need to shed light on the details of these relationships.

## Conclusion

Although the mTOR signaling pathway is mainly known for its role as a regulator of energy metabolism, its balanced activity has a major impact on healthy development and homeostasis in the brain. Due to its multiple interactions with different receptors, ligands, and other signaling pathways, the mTOR pathway is a central and crucial signaling pathway affecting numerous functions in astrocytes; however, its physiological functions and regulation awaits further investigation. In pathophysiology, dysregulated mTOR signaling not only affects the morphology, metabolism and functionality of individual cell types in the brain, it also significantly influences the development, morphology and functionality of the CNS. This often results in serious neurological disorders, such as epilepsy and brain tumors. A better understanding of the influence of this signaling pathway on functions of astrocytes and other brain cell types will contribute to a better understanding of brain physiology and brain disorders and might provide the basis for the development of novel therapy approaches.

## Data Availability

No datasets were generated or analysed during the current study.
